# In Vivo Targeting of the Neurovascular Unit: Challenges and Advancements

**DOI:** 10.1007/s10571-021-01113-3

**Published:** 2021-06-04

**Authors:** Oandy Naranjo, Olivia Osborne, Silvia Torices, Michal Toborek

**Affiliations:** 1 Department of Biochemistry and Molecular Biology, University of Miami Miller School of Medicine, Miami, FL 33136, USA; 2 Institute of Physiotherapy and Health Sciences, The Jerzy Kukuczka Academy of Physical Education, Katowice, Poland; 3 Department of Biochemistry and Molecular Biology, University of Miami School of Medicine, Gautier Bldg., Room 528, 1011 NW 15th Street, Miami, FL 33136, USA

**Keywords:** Neurovascular unit, Imaging, Protein expression, Cre mouse models, Blood–brain barrier

## Abstract

The blood–brain barrier (BBB) is essential for the homeostasis of the central nervous system (CNS). Functions of the BBB are performed by the neurovascular unit (NVU), which consists of endothelial cells, pericytes, astrocytes, microglia, basement membrane, and neurons. NVU cells interact closely and together are responsible for neurovascular coupling, BBB integrity, and transendothelial fluid transport. Studies have shown that NVU dysfunction is implicated in several acute and chronic neurological diseases, including Alzheimer’s disease, multiple sclerosis, and stroke. The mechanisms of NVU disruption remain poorly understood, partially due to difficulties in selective targeting of NVU cells. In this review, we discuss the relative merits of available protein markers and drivers of the NVU along with recent advancements that have been made in the field to increase efficiency and specificity of NVU research.

## Introduction

**Fig. 1:**
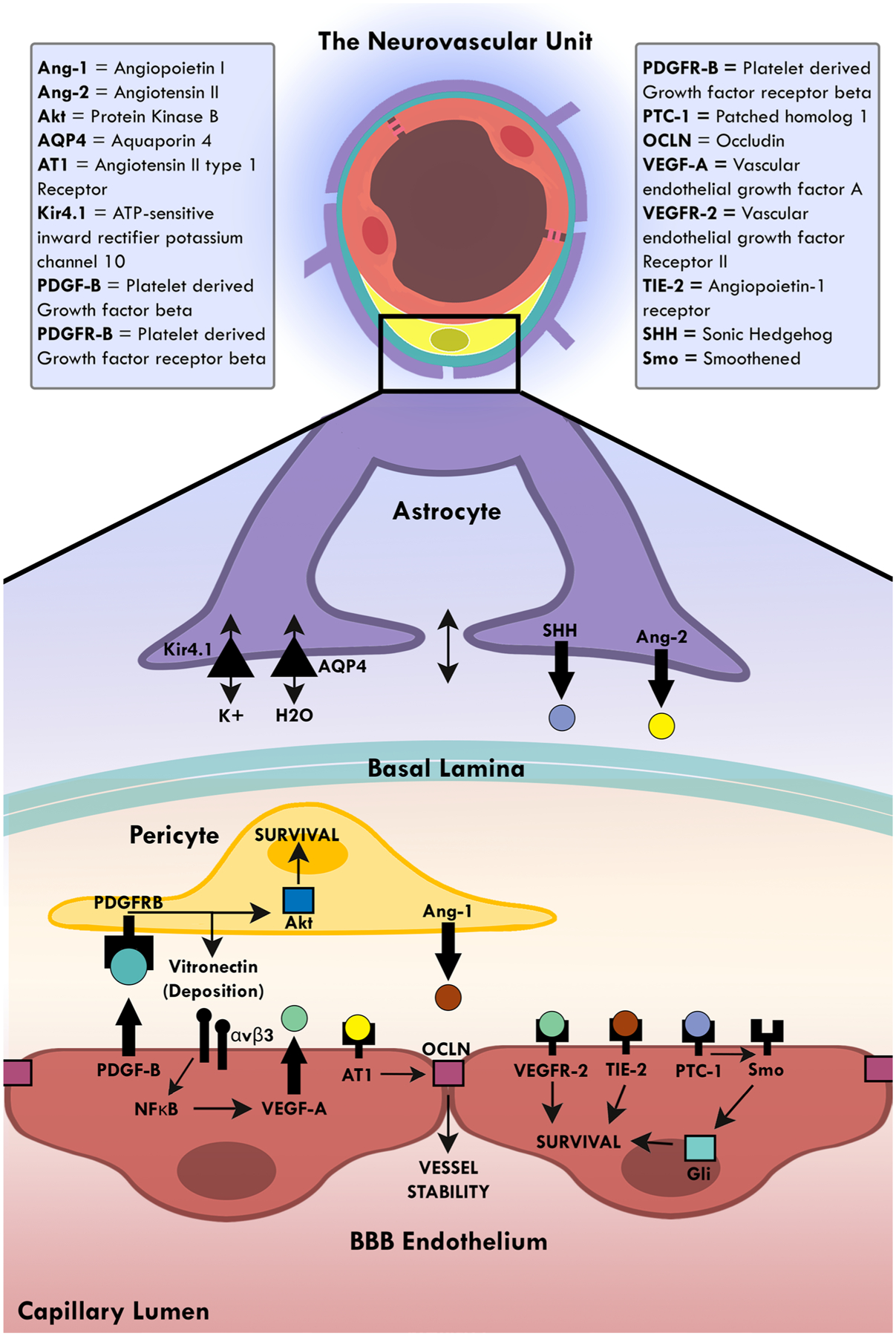
Crosstalk at the neurovascular unit. A schematic diagram illustrating selective interactions between endothelial cells, pericytes, and astrocytes that maintain the blood brain barrier (BBB). The cells of the neurovascular unit provide the mature endothelium with the survival signals required for maintaining the functional BBB. Pericytes secrete angiopoietin I and vitronectin which maintain healthy endothelial cells. Endothelial cells secrete platelet-derived growth factor-beta that maintain survival of pericytes. Astrocytes secrete Sonic Hedgehog which functions through the patched receptor and lead to survival signals in endothelial cells. Astrocytes also control the BBB microenvironment and permeability. Through angiotensin II expression, astrocytes influence occludin expression in tight junctions and barrier properties. Lastly, astrocytes can change ionic concentrations through the water and potassium channels, such as aquaporin 4 and ATP-sensitive inward rectifier potassium channel 10

Communication between the cells of the neurovascular unit (NVU) that create and maintain the framework of the blood–brain barrier (BBB) is critical to the homeostasis and proper function of the central nervous system (CNS). Endothelial cells, pericytes, astrocytic end feet, and microglia closely interact with each other to form the basis of the NVU and the BBB. Their communicative signals act in concert to ensure proper regulation. For example, endothelial cells rely on vitronectin and angiopoietin I from pericytes, and Sonic Hedgehog from astrocytes (Hill et al. 2019). While all three of these ligands function through different pathways, their mutual cascade results in endothelial cell survival and BBB maintenance. In return, endothelial cells secrete platelet-derived growth factor-beta (PDGF-β), which provides reciprocal survival signals to pericytes through protein kinase B and stimulates further vitronectin production (Chatterjee and Naik 2012). Astrocytes of the NVU express angiotensin II, which contributes to vessel stability through modulation of occludin tight junctions, an essential protein for BBB permeability (Wosik et al. 2007). Lastly, astrocytes are critical to the microenvironment of the NVU, controlling ion and water concentrations through aquaporin 4 on the cell membranes of astrocytic endfeet that interact with endothelial cells (Abbott et al. 2006) (Fig. 1). While communication between these cells is essential, certain neurological pathologies can induce signal attenuation via BBB breakdown.

Common neuropathologies that are induced by BBB breakdown and subsequent NVU signaling disruption include Alzheimer’s disease (AD), multiple sclerosis (MS), and stroke. Disruption of NVU stability leads to dysfunction of the BBB which has been increasingly linked to pathobiology of neurocognitive disorders, CNS infections, and toxicity of substance abuse (Bertrand et al. 2019a, b; Castro et al. 2016; Cho et al. 2017; Daneman et al. 2010; Kisler et al. 2020; Lee et al. 2001; Mathiisen et al. 2010; Nakagawa et al. 2012; Zlokovic 2008). For example, the breakdown of the BBB is a central factor to the onset and progression of AD (Zlokovic 2010). One of the leading hypotheses in AD research is that pathological accumulation of beta-amyloid (Aβ) leads to plaque formation over time. Aβ plaque deposits in both the brain parenchyma and cerebral blood vessels lead to various vessel pathologies including decreased cerebral blood flow and BBB breakdown (Merlini et al. 2011). Research has shown that activation of both microglia and astrocytes are critical to AD pathology and both cell types play a role in Aβ protein concentration and clearance, respectively (Fuhrmann et al. 2010; Liu et al. 2010; Merlini et al. 2011). Moreover, a loss of capillary pericytes is observed in the white matter of AD patients and other common dementias (Ding et al. 2014). Therefore, identifying specific markers for these cell types may aid in rapid diagnostic, provide tools for better understanding of the disease process as well as therapeutic assessments of AD patients.

MS is a neuroinflammatory disease whereby resident and infiltrating immune cells form lesions in the white matter of the CNS that lead to demyelinating plaques (Lassmann 2010). Evidence suggests that BBB disruption through endothelial cell dysfunction and the release of inflammatory cytokines is observed early on in disease progression (Correale et al. 2017; Minagar and Alexander 2003). T cells, microglia, monocytes, and fibrinogen proteins all contribute to the neuroinflammation observed (Adams et al. 2007; Ajami et al. 2011; Heppner et al. 2005). While the progression of the disease is still poorly understood, evidence suggests that targeting the cells of the NVU is critical for a mechanistic understanding of MS and how to treat it. For instance, attenuating BBB dysfunction through endothelial wnt/β-catenin signaling reduces immune cell infiltration and improves neurological condition (Lengfeld et al. 2017). Retinoic acid expression in pericytes was found to modulate the endothelial wnt/β-catenin signaling pathway (Lai et al. 2003), which offers a potential target for MS therapy. By utilizing NVU markers, we can better characterize the breakdown of the BBB that leads to CNS infiltration by immune cells, providing potential new therapeutic targets.

Similarly, one of the hallmarks of ischemic stroke pathology is the breakdown of cerebrovasculature and the BBB, which increases paracellular permeability. Despite better treatments, imaging techniques, and understanding of stroke-associated risk factors, diagnosis of acute stroke remains difficult (Arch et al. 2016). In actuality, studies show that about 25% of ischemic stroke patients are difficult or impossible to diagnose, therefore novel strategies are needed to diagnose within a therapeutic window (Amarenco et al. 2009). Given the small therapeutic window of ischemic stroke, timely diagnosis can significantly improve post-stroke outcomes and lower stroke mortality (Jahan et al. 2019). Recent work in the cerebrovascular field shows that biomarker panels rather than a single marker can be an effective approach to stroke diagnosis; however, their clinical accuracy has yet to be confirmed (Jickling and Sharp 2015). In order to assess marker levels, we must understand the signaling pathway progression during ischemic onset, and more specifically, the cells of the NVU that secrete distinct signals during acute, subacute, and late phases of stroke (Steliga et al. 2020). Therefore, diagnostic markers for BBB dysfunction that can efficiently target NVU cells at different timepoints may be critical to identify stage-specific biomarkers and understand the changes in intercellular communication within the NVU during brain pathologies.

Progress on the NVU involvement in neuropathology has been hampered due to lack of specific imaging and targeting techniques as well as heterogeneity within NVU cell populations (Smyth et al. 2018; Yoon et al. 2017). Moreover, targeting techniques within live systems are exceedingly difficult. The main challenge not only lies in identifying NVU markers for the generation of in vivo models, but also the ability and limitations in linking the marker etiologies during cerebral abnormalities to pathophysiology and histopathology. While there has been a growing list of drivers and markers for NVU cells, they frequently lack full specificity, resulting in cross reactivity with other cells. However, even semi-specific markers can be a powerful tool for NVU experiments if the limitations are understood when interpreting the data. Likewise, work has been done to produce novel Cre genetic mouse models and drive expression of specific proteins in the cells of the NVU. This review discusses current standard markers of NVU cells and their pitfalls, along with the new advancements in the field that aid in the design of mechanistic in vivo studies evaluating the involvement of the NVU in the pathobiology of neurological diseases.

## Endothelial Cells

Endothelial cells originate from mesoderm and line blood and lymphatic vessels throughout the body (Bautch and Caron 2015). They constitute the main cellular element of the BBB by forming tight junctions that link endothelial cells together and form a selectively permeable barrier between the blood and the CNS. Ultimately, the BBB maintains the homeostasis of the CNS and prevents toxic molecules, viruses, bacteria, and inflammatory cells from reaching the brain (Bernacki et al. 2008; Daneman and Prat 2015). Visualizing endothelial cells is crucial to understanding how different experimental conditions can affect BBB permeability.

CD31, CD34, and CD54 are all standard markers for the vascular endothelium (Table 1), but they lack full selectivity as they are also expressed in other cell types (Pusztaszeri et al. 2006). For example, CD31, known as platelet/endothelial cell adhesion molecule 1 (PECAM-1), is also expressed in B cells, platelets, macrophages, monocytes, NK cells, and T cells (Nourshargh et al. 2006). CD34 expresses cross reactivity with dendritic cells, hematopoietic stem cells, leukemic cells, and endothelial progenitor cells (Sidney et al. 2014). In addition to endothelial cells, CD54, known as intercellular adhesion molecule 1 (ICAM-1), is expressed in lymphocytes and macrophages (Dustin et al. 2011). Nevertheless, even with significant cross reactivity, CD31, CD34, and CD54 can serve as effective markers in immunostaining studies because endothelial vasculature is visually different from other cell types. VE-cadherin, known also as Cadherin 5 (Cdh5), is another effective marker for vascular endothelium. Cdh5 protein is found at endothelial cell junctions where it plays a critical role in endothelial barrier function and angiogenesis (Dejana et al. 2009). Cdh5 is specific for endothelial cells at the NVU; however, it can also be expressed on hematopoietic stem cells and mesenchymal stem cells (Vestweber 2008).

A marker with very high specificity for the vascular endothelium is Lycopersicon esculentum (Tomato) lectin (LEL) (Mazzetti et al. 2004; Smolkova et al. 2001). Lectins conjugated to different dyes are commercially available and can be employed to visualize vasculature using intravascular perfusion methods or direct application to tissue sections (Nikolakopoulou et al. 2017; Robertson et al. 2015). LEL binds to sugars enriched on the basement membrane of endothelial cells in human and rodent samples (Piller et al. 1990). The advantages of LEL are that it does not depend on primary and secondary species-specific antibody interactions. Angiopoietin-1 receptor (TIE-2) is a surface receptor that regulates angiogenesis and cell survival. TIE-2 is expressed in both young and adult endothelial cells but also on a subset of angiogenic promoting macrophages (De Palma et al. 2005). In the neurovascular unit, TIE-2 is very specific to endothelial cells and can be used as a target for endothelial visualization (Fukuhara et al. 2009; Sato et al. 1995). Lastly, vascular cell adhesion protein 1 (VCAM-1/CD106) can modulate BBB endothelium to increase permeability to immune cells and is activated upon proinflammatory stimulation (Haarmann et al. 2015). VCAM-1 can be used as a marker to visualize injury and inflammation in endothelial cells of the BBB; however, it is also expressed by tissue macrophages, dendritic cells, bone marrow fibroblasts, myoblasts, oocytes, Kupffer cells, and Sertoli cells (Kong et al. 2018; Osborn et al. 1989).

## Drivers for Endothelial Cell Expression

Cre recombinase mouse models are a powerful tool for targeted protein expression. Constitutive and inducible Cre models are based on a cell specific promoter driving Cre expression. CreERT2 encoding a Cre recombinase (Cre) fused to a mutant estrogen ligand-binding domain (ERT2) is a widely used Cre-ER version, which has low background Cre activity and robust activation following tamoxifen treatment (Table 2). Tek-Cre (Tie2-Cre) was the first endothelial specific Cre mouse model and still the most commonly used (Payne et al. 2018). Tie-2 Cre models are characterized by constitutive expression in all endothelial cells starting from early development and continuing into adulthood. Because Tie-2 is expressed by a precursor of both blood and endothelial lineage, Cre recombinase activity in Tie-2 Cre models are also present in cells of hematopoietic lineage (Kisanuki et al. 2001; Tang et al. 2010).

VE Cadherin/Cadherin 5 (Cdh5-Cre/ERT2) is frequently employed as an inducible driver that is specific for endothelial cells. Cdh5-Cre/ERT2 uses three Cadherin 5 alleles in the Cdh5 locus to drive Cre/ERT2 expression (Monvoisin et al. 2006; Okabe et al. 2014; Wang et al. 2010). More specifically, the Tg(Cdh5-cre/ERT2)^1Rha^ allele has been published for pan-endothelial expression in retina and brain endothelium of post-natal mice (Wang et al. 2010; Yanagida et al. 2017). Two alleles, Tg(Cdh5-cre/ERT2)^#Ykub^ and Tg(Cdh5-cre/ERT2)^CIVE23Mlia^, have been well characterized in pre-natal mice. One aspect of Cre recombinase models to note is their potential for experimental variability in recombination efficiency. Variability in the Cdh5 inducible allele expression has been reported with identical tamoxifen doses and between different floxed alleles within the same mouse (Zarkada et al. 2015). This variability, given the same amount of tamoxifen, poses as a potential weakness in the model system and should be accounted for.

**Fig. 2:**
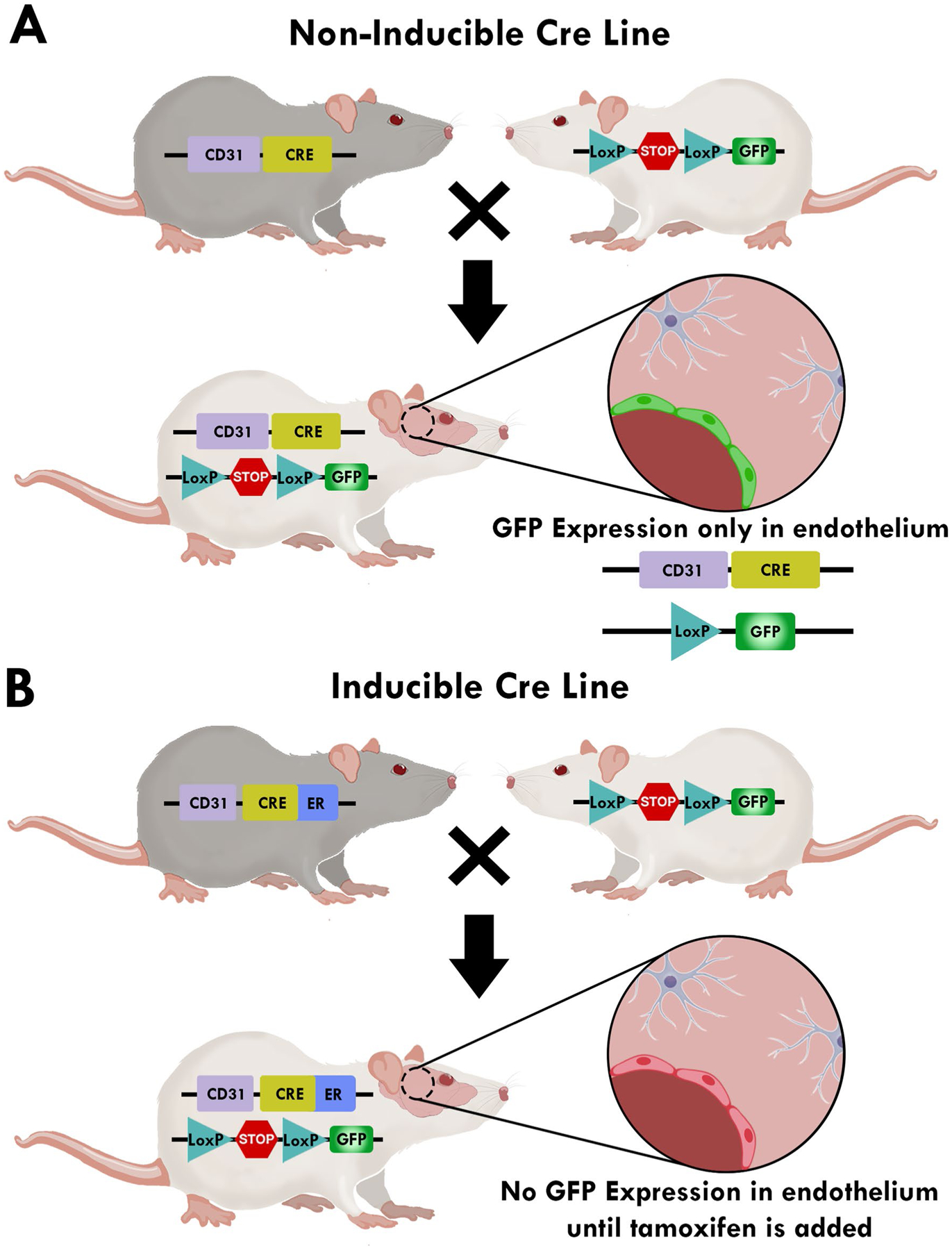
Schematic of non-inducible and inducible Cre lines. **A** Non-inducible Cre lines depend solely on the activity of their cell specific promoter. When both mouse lines are crossed Cre recombinase functions to excise the stop codon in front of green fluorescent protein (GFP) which turns GFP production on in cells where CD31 is active, in this example it is endothelial cells. **B** In an inducible Cre line, Cre is fused to mutated hormone-binding domains of the estrogen receptor. Cre-ER is inactive until tamoxifen is added. Inducible lines allow for both spatial and temporal control by combining the tissue-specific expression of Cre-ER by CD31 and its tamoxifen-dependent activity of the enzyme

Figure 2 provides examples of generating mice expressing GFP in the vascular endothelium using CD31 as endothelial cell driver. In this model, CD31 drives Cre recombinase then excises the stop codon flanked by loxP sequences. With no stop codon, GFP can be expressed in any cell with CD31 activity (Fig. 2A). An inducible Cre model can be achieved by fusing Cre recombinase with a ligand-binding domain of the human estrogen receptor (ER) as a transgene, resulting in tamoxifen-dependent Cre recombinase. Under these conditions GFP is expressed in CD31 positive cells until the animal receives a tamoxifen regimen, upon which tamoxifen metabolite 4-hydroxytamoxifen (4-OHT) activates ER (Fig. 2B).

## Brain Pericytes

Brain pericytes are mural cells that cover brain capillaries and venules with their cytoplasmic processes close to 100%. Pericytes are highly heterogeneous, which may reflect their origin that varies in different parts of the body and even in various regions of the brain. While pericytes in the forebrain are derived from neural crest cells, pericytes in the rest of the brain appear to originate from mesenchymal stem cells of the mesoderm (Zheng et al. 2020). Moreover, recent evidence indicates that myeloid progenitors also contribute to the developing of pericytes in the brain. In fact, it was proposed that a substantial pool of brain pericytes originate from yolk-sac-derived macrophage progenitors (Yamazaki et al. 2017). Developmentally, pericyte recruitment to CNS vessels (E11 in rat cerebral cortex) correlates with the onset of barrier properties of the BBB (Daneman et al. 2010). They are the only cell in the NVU to have direct contact with BBB endothelium as part of pericyte–endothelium interface, called peg-socket contacts, that lacks a basement membrane. Pericytes are responsible for regulation of paracellular/transendothelial fluid transport, homeostasis of the microenvironment, and protecting endothelial cells from toxic substances (Armulik et al. 2011; Daneman et al. 2010; Nikolakopoulou et al. 2019).

Interestingly, pericytes remain a relatively poorly defined cell type due to the lack of highly specific cell markers. The difficulty of targeting this cell type due to their heterogeneity. In addition, their marker expression depends on anatomical location and function (Attwell et al. 2016). Because there is no pan-pericyte specific cell marker, morphology and a combination of at least two cell markers are necessary to identify pericytes in the brain (Smyth et al. 2018). Platelet-derived growth factor receptor-β (PDGFRβ) (Table 1) is the most commonly used marker for pericyte identification. PDGFRβ/PDGFβ signaling is essential for pericyte maintenance and loss of that signaling leads to a progressive depletion of pericytes in vivo (Daneman et al. 2010; Nikolakopoulou et al. 2017). However, PDGFRβ is not fully specific for pericytes as it is also expressed in neural progenitor cells, glia, and fibroblast cells during injury (Kyyriainen et al. 2017). Chondroitin sulfate proteoglycan 4/neural glial antigen 2 (NG2) and aminopeptidase N (CD13) are the two most common markers co-stained with PDGFRβ to confirm pericyte identification (Hartmann et al. 2015). NG2 is expressed on the cell surface of pericytes during angiogenesis (Ozerdem et al. 2001) but also in oligodendrocyte progenitors, Schwann cells, and perineurial cells (Levine et al. 2001; Stallcup 2002). CD13 is a membrane bound protein originally identified as a marker for cells of myeloid origin and can be used as a surface marker for brain pericytes (Armulik et al. 2010). Melanoma cell adhesion molecule (CD146) has been used as a marker for pericytes of the NVU due to its involvement in PDGFRβ activation and BBB integrity (Chen et al. 2017; Patenaude et al. 2015). CD146 is expressed by endothelial cells in BBB development but not in mature vessels, ganglion cells, and activated T lymphocytes (Chen et al. 2017; Nodomi et al. 2016; Shih 1999; Wang and Yan 2013). Pericytes are also positive for neuroectodermal stem cell marker (nestin). Nestin is a type VI intermediate filament that is generally used for the identification of neural stem cells. Expression of nestin is higher in other regions of the brain and also present in endothelial cells adjacent to pericytes (Klein et al. 2014). Recent studies show that a FluoroNissl dye (NeuroTrace 500/525) can accurately label capillary pericytes in the live mouse brain. Normally this dye is used to label neurons in fixed tissue but when applied to living cells there is robust overlapping expression with endogenous PDGFR-β and NG2-positive cells on brain capillaries. NeuroTrace 500/525 can be used to visualize pericytes in live imaging of the mouse brain (Damisah et al. 2017). α-smooth muscle actin (SMA) has been omitted from the list because of ongoing debate on whether pericytes express SMA. SMA specificity for pericytes in vivo is unknown, with little evidence showing pericytes express SMA in vitro.

A growing availability and efficiency of RNA-Sequencing has led to the development of new gene expression libraries. A review of five RNA-Seq libraries for brain mural cells identified 260 genes that are significantly enriched in mural cells and can therefore be used to specifically identify pericytes (He et al. 2018). The list was validated by evaluating immunofluorescence imaging of brains stained with for vitronectin (high expression) and interferon reduced transmembrane protein 1 (low expression). Analysis of these markers showed enriched expression in pericytes and expected low levels of expression in other CNS cells.

## Drivers for Pericyte Expression

PDGFRβ-Cre mouse models have been the most common way of driving protein expression in pericytes. PDGFRβ-Cre models are available in constitutively active (Cuttler et al. 2011; Foo et al. 2006) and inducible forms (Cuttler et al. 2011; Gerl et al. 2015; Kruse et al. 2019). The inducible PDGFRβ-P2A-CreER^T2^ model has been validated with coimmunostaining and time course experiments to test pericyte expression and has high levels of Cre recombinase in pericytes of the retina and brain (Cuervo et al. 2017). While PDGFRβ-Cre mouse models work well, PDGFRβ alone is far from being specific to pericytes (Sweeney et al. 2016). As a driver alone, PDGFRβ can lead to unwanted expression in other cell types that express this marker protein and were described above. Therefore, a two-driver promoter model can increase specificity of pericyte responses. A recent advancement in the field is the creation of a PDGFRβ/NG2 inducible mouse model (Kisler et al. 2020), which is consistent with the immunofluorescence approach of using at least two markers for pericyte identification. The pericyte-CreER model has flp recombinase under the control of PDGFRβ and a stop cassette under the control of the NG2 promoter (Nikolakopoulou et al. 2019). After tamoxifen injection only cells that have both an active NG2 and PDGFRβ will express the protein of interest.

## Astrocytes

Astrocytes are specialized glial cells that originate from progenitor cells in the neuroepithelium of the developing nervous system (Rowitch and Kriegstein 2010; Tabata 2015). They form a glial network that ensheathe both neurons and blood vessels of the CNS (Vasile et al. 2017). Their connection between blood vessels and neurons allows for neurovascular coupling which includes the dilation/contraction of smooth muscle cells that surround vessels (Haydon and Carmignoto 2006). Research has shown that they have an active role in information processing and BBB control, making astrocytes an important cell to understand neurological diseases (Dossi et al. 2018; Guillamon-Vivancos et al. 2015). Astrocytes are not a homogenous population and therefore no single and fully specific marker exists to target all of them (Yoon et al. 2017). Glial fibrillary acidic protein (GFAP) (Table 1) is an intermediate filament expressed by reactive astrocytes, progenitor cells (Garcia et al. 2004; Zhang and Jiao 2015) and fibroblasts (Hainfellner et al. 2001; Sofroniew and Vinters 2010). Interestingly, GFAP-positive progenitor cells contribute to the development of vascular smooth muscle cells and endothelial cells (Osman et al. 2020). In the peripheral nervous system, GFAP-positive staining has been demonstrated in Schwann cells, enteric glial cells, and satellite cells of human sensory ganglia. As the most common marker for astrocyte cell body visualization, GFAP has been well categorized use in vivo and in vitro (Garcia et al. 2004; Zhuo et al. 2001). While it is uniformly expressed by astrocytes in cultures, in vivo GFAP has been shown to favor white matter astrocytes (Lundgaard et al. 2014) with only about 15% of the total astrocyte population showing positive staining (Bushong et al. 2002; Cahoy et al. 2008). One of the reasons for this selective staining may be due to GFAP upregulation in reactive astrocytes (Sofroniew and Vinters 2010). Astrocytes in the corpus callosum, cerebral peduncle, and especially the hippocampus preferentially express GFAP (Zhang et al. 2019).

Another marker for staining of astrocytes is aldehyde dehydrogenase 1 family member L1 (ALDH1L1) which is expressed in astrocytes but also in neural stem cells (Foo and Dougherty 2013). ALDH1L1 is highly specific and more widely expressed across the astrocyte population compared to GFAP (Cahoy et al. 2008). Even though ALDH1L1 is more inclusive than GFAP, the antibodies are reported to be difficult to work with, leaving GFAP as the most commonly used astrocyte marker. Glutamate transporters, such as excitatory amino acid transporter 1 (EAAT1 or glutamate aspartate transporter 1, GLAST1) and EAAT2 (GLAST 2), are present on astrocyte membranes and are in higher concentrations in astrocytes near capillaries or neuronal synapses (Parkin et al. 2018). Due to their location, EAAT1 and 2 can be used as regional markers of astrocytes. However, they can also be found in microglia, and oligodendrocytes, even though in lower concentrations (Kondo et al. 1995; Parkin et al. 2018). Aquaporin 4 (AQP4) is the predominant water channel expressed in astrocytes and can be used to visualize astrocytic end feet (Michalski et al. 2017). AQP4 is highly expressed in reactive astrocytes but also in microglia (Ikeshima-Kataoka 2016). Calcium binding protein B (S100β) is a glial specific protein that can be used to visualize a subset of mature astrocytes that ensheathe blood vessels (Raponi et al. 2007). In contrast to GFAP, S100β is a more suitable marker to target all astrocytes but is particularly good to target astrocytes of the cortex and thalamus (Zhang et al. 2019). S100β is also expressed by oligodendrocytes, oligodendrocyte progenitor cells, and microglia (Cahoy et al. 2008; Raponi et al. 2007). Plasma levels of soluble S100β are also used as a marker of BBB disruption (Blyth et al. 2009; Koh and Lee 2014). Aldolase C (Aldoc) has been shown to localize in astrocytes of the cerebrum, Purkinje cells in the cerebellum, and large gray matter neurons. Aldoc is not usually used as an astrocyte biomarker due to lack of specificity (Fujita et al. 2014).

## Drivers for Astrocyte Expression

GFAP and ALDH1L1 are the most specific genetic drivers for astrocytes. Depending on the brain region, GFAP Cre mouse model (hGFAP-CreERT2xAi14) has been characterized to have between 50 and 89% coverage of astrocytes, with specificity for astrocytes ranging between 52 and 92%. Characterizations of different GFAP Cre-strains have shown significantly less coverage and specificity in the cortex, suggesting that specificity and coverage are also dependent on the strain and method of induction (Park et al. 2018). In contrast, Aldh1L1-Cre/ERT2 mice have very high levels of expression in astrocytes, and no detectable expression in neurons (Winchenbach et al. 2016). Both Cre lines can be crossed to any desired proteins of interest, which are usually available through commercial methods, making Cre mouse models a useful genetic tool.

## Microglia

Microglia are glial cells located throughout the brain and spinal cord and they serve as the main form of immune defense for the CNS (Li and Barres 2018). During insult to the CNS, microglia transform from a resting to an activated state and their gene expression changes to perform their immune role (Cronk and Kipnis 2013). The heterogeneity and plasticity of microglia make them a difficult cell to target and must be considered in the selection of effective markers. A set of common markers used for microglia are integrin alpha M subunit (CD11b) (Gong et al. 2019), Ionized calcium binding adaptor molecule 1 (Iba1) (Burkovetskaya et al. 2020), CX3C chemokine receptor 1 (CX3CR1) (Chen et al. 2002), and CD45 (Majerova et al. 2019). All these markers can be used to distinguish microglia from other cells of the CNS but not from peripheral immune cells in the meningeal spaces or perivascular macrophages in the perivascular spaces. A few promising discoveries made in the field indicated that Transforming growth factor β (TGFβ)-dependent signaling might be useful in distinguishing microglia from macrophages (Butovsky et al. 2014). Proteins related to the TGFβ pathway, such as transmembrane protein 119 (Tmem119) (Satoh et al. 2016), purinergic receptor (P2RY12) (Amadio et al. 2014; Haynes et al. 2006), Fc Receptor like S (FCRLS) (Butovsky and Weiner 2018), and hexosaminidase subunit beta (HexB) (Butovsky et al. 2014), function as stably expressed microglial-specific markers. The function of Tmem119 is not fully understood; however, its expression patterns show strong levels of expression in microglia (Butovsky et al. 2014; Butovsky and Weiner 2018). Tmem119 has been studied under a variety of disease conditions (e.g., sciatic nerve injury-induced microglial activation, lipopolysaccharide-induced systemic inflammation, optic nerve crush injury, Alzheimer’s disease, or multiple sclerosis) and was shown to maintain stable but low expression in activated microglia (Bennett et al. 2016).

P2RY12 is found on the surface of blood platelets, where it is an important clotting regulator (Dorsam and Kunapuli 2004). However, its expression in the CNS appears to be specific to microglia and is necessary for microglial functions, including neuronal monitoring and neuroprotection (Cserep et al. 2020). In contrast to Tmem119, P2RY12 expression is enhanced by anti-inflammatory cytokines, which may make it a better marker for microglia. Overall, P2RY12 is regarded as one of the most specific available markers for yolk-sac derived microglia (Jurga et al. 2020).

Among other potential microglial markers, FCRLS is highly active in rodent microglia; however, there is no known expression of this gene in human microglia. Gene expression shows high and stable levels of FCRLS expression in isolated microglia and no expression in macrophages (Butovsky et al. 2014). Hexb is an enzyme responsible for the degradation of ganglioside and molecules containing terminal N-acetyl hexosamine (Kyrkanides et al. 2012). Hexb is required for microglial maintenance and while it has not been fully studied in a variety of pathological conditions, it maintains stable expression in several neurodegenerative and neuroinflammatory conditions (Butovsky et al. 2014).

In studies of neuroinflammation it is not only important to distinguish microglia and macrophages but also resting and active microglia. Microglia are the resident immune cell, and therefore are equipped with tools to recognize and repair pathological conditions. Proteins associated with MHC complexes, Inflammatory signal production and release, Toll-like receptors (TLRs), and phagocytosis can be used as markers for microglial activation (Boche et al. 2013). Examples of such proteins are CD16 (Kigerl et al. 2009), CD32 (Kigerl et al. 2009), Major histocompatibility complex II (MHC II) (Smith and Dragunow 2014), and CD163 (Etzerodt et al. 2014); however, there are many markers that can be used for microglial activation (Jurga et al. 2020). These markers are upregulated during pathological conditions and when used together with generic microglial markers could altogether distinguish resting and active microglia.

## Drivers for Microglial Expression

CX3CR1-Cre reporter mice have been the most common animal model for microglial expression (Jung et al. 2000; Wieghofer and Prinz 2016). These mice are available in both inducible and constitutively active forms (Fig. 2). Reporter expression can be found in monocytes, dendritic cells, NK cells, as well as T and B lymphocytes (Goldmann et al. 2016). However, there have been valid concerns over the leakiness (to varying degrees and depending on the specific line) of CX3CR1-Cre GFP reporter lines in neurons. Furthermore, CX3CR1 cannot be used to distinguish the effects of microglia vs. infiltrating macrophages, which is critical to understanding the role of microglia in health and disease (Jurga et al. 2020). Instead, Tmem119-EGFP (Kaiser and Feng 2019), Tmem119-CreERT2 (Kaiser and Feng 2019), P2RY12-CreER (McKinsey et al. 2020), and Hexb-CreERT2 (Masuda et al. 2020) are promising and microglia-specific models that have been developed and published in the last 2 years. Tmem119-EGFP and Tmem119-Cre-ERT2 were generated using donor DNA from peptide porcine teschovirus-1 polyprotein and EGFP (P2A-EGFP) or CreERT2 (P2A-CreERT2) along with CRISPR Cas9 into C57BL/B6N zygotes. Strains were validated by PCR products and confirmed specific insertion of the construct into the Tmem119 locus. Using confocal microscopy for both the inducible and non-inducible lines, GFP expression was confirmed in microglia but not perivascular, meningeal or choroid plexus macrophages. Unfortunately, the lines were not studied under pathological conditions in which Tmem119-related expression is expected to decrease.

P2RY12-CreER is an inducible Cre line based on Cre and the estrogen receptor fusion protein. P2RY12 is considered to be one of the most specific microglial markers. While its expression was observed in a subset of macrophages in the dura matter, it was absent in macrophages or circulating monocytes. Outside of the CNS, P2RY12 expression was observed in subsets of CD206+ Cx3cr1+ cells in the heart, intestine, lung, and spleen as well as in LYVE1+ cells. P2RY12-CreER models were tested in disease models of acute ischemic stroke and multiple sclerosis, and it was demonstrated that P2RY12 expression was maintained in areas of injury.

Hexb-tdT a reporter line and Hexb-CreERT2 a line for Cre inducible expression is based on HexB, which is a stable core microglial protein. Hexb-CreERT2 was studied in the 5xFAD mouse model of Alzheimer’s disease and no Hexb expression associated with macrophages was observed in the CNS. The reported advantages of Hexb is that there is little to no downregulation during inflammatory conditions in comparison to P2RY12 and Tmem119. Moreover, Hexb expression is enhanced in parenchymal microglia than other models for microglial targeting.

## Conclusions and Advancements/Perspectives of the Field

Formed by neurons, endothelial cells, pericytes, astrocytes, microglia, and the extracellular matrix, the NVU controls and regulates BBB integrity and cerebral blood flow. To maintain the BBB, various cell types of the NVU communicate and develop individual tasks (Kisler et al. 2017; Muoio et al. 2014). Dysfunction of the BBB is associated with multiple neurological disorders for which an optimal identification of the NVU subtypes is essential for understanding the underlying pathology. Currently, the markers and drivers available to identify cells of the NVU can lead to a cross reactivity, limiting the interpretation of the findings. Several new approaches are successfully being developed to solve this residual challenge.

Cre models are a key method for spatial and temporal control of gene expression and protein targeting. Some pitfalls in Cre systems is that there is a permanent modification to the genome induced by Cre and their dependency on available tissue-specific markers to drive protein expression. To solve these problems, this system is in constant improvement. One example of the extraordinary level of sophistication that is being achieved is the use of more than one driver for an increase in cell specificity. As an example, pericyte-CreER mentioned above crosses NG2 and PDGFRβ, which increases specificity for pericytes. Double promoter models allow for more advanced animal models targeting the NVU. Another recent advancement in the field is the possibility of generating a model that includes both, a specific target, and reversible transgenic misexpression with spaciotemporal resolution. With this model, gene expression depends on the intersection of previous Cre exposure and (r) tTA activity (Rosello-Diez et al. 2018). An example of the possibilities that this technology provides is the development of the intersectional *Dragon*-DTA model (Ahmadzadeh et al. 2020), which allows for acute tissue-specific cell ablation with spatiotemporal exactitude. With a growing number of commercially available Cre models and techniques, there are many possibilities to generate more advanced mouse lines to target the NVU.

Additionally, we can learn a lot from how other fields solve the problem of in vivo targeting. In the cancer field the use of lentiviruses to recognize cancerous tissue is becoming an important development. Lentiviruses can recognize and deliver a plasmid to tumor cells (Russell and Peng 2007). The viruses are manipulated to take advantage of cancer morphology and aberrant signaling so that viral replication is only viable in a cancer cell (Bell et al. 2003). While the brain represents a challenging target for gene delivery, certain viruses have already proved capable of preferential delivery to CNS cells. Adeno-associated virus serotype 2 (AAV2) infects neurons preferentially, while AAV5 efficiently transduces Purkinje cells, but not granule cells (Bartlett et al. 1998; Haberman et al. 2002). Among AAVs, AAV9 has the highest penetration through the BBB (Saraiva et al. 2016). Targeting the neurovascular unit could be a matter of finding the right serotype for the cell or manipulating viral vectors to create an environment for specific gene delivery.

Finally, the growing understanding and availability of nanoparticles offers another way of studying the NVU. Their small size and use of biodegradable materials make nanoparticles a unique system for targeted delivery into cells or tissues (Thurn et al. 2007). For example, glucose-coated gold nanoparticles can cross endothelium via passive uptake and concentrate in astrocytes in vivo (Gromnicova et al. 2013). Nanotechnology also allows for drugs to effectively cross the BBB via transient pathways without altering vascular and neurological integrity (Ding et al. 2014; Osborne et al. 2020; Yamaguchi et al. 2020; Zhou et al. 2018). A growing ability to manipulate the properties of nanoparticles can prove to be a great advantage in targeting NVU cells.

Here we summarize the current knowledge concerning the markers and drivers of the NVU. To better understand the NVU and its role in brain pathology, we must explore and be able to specifically identify and manipulate different NVU cell types. New technologies to approach the problem are being developed and will help us better understand the molecular basis of the BBB and neurovascular interactions in health and neurological disorders.

## Figures and Tables

**Table 1:** Cell markers of the neurovascular unit

Endothelial cells	
Marker	Non-endothelial expression
CD31/PECAM-1	B cells, platelets, macrophages, monocytes, natural killer cells, and T cells
CD34	Dendritic cells, hematopoietic stem cells, leukemic cells, and endothelial progenitor cells
CD54/ICAM-1	Lymphocytes and macrophages
VE-Cadherin/Cdh5	Hematopoietic and mesenchymal stem cells
Lectin	No reported expression
Tie-2	Subset of macrophages
CD106/VCAM-1	Tissue macrophages, dendritic cells, bone marrow fibroblasts, myoblasts, oocytes, Kupffer cells, and Sertoli cells
Pericytes	
Marker	Non-pericyte expression
PDGFRβ	Neural progenitor cells, glia, and fibroblast cells during injury
CD13	Cells of myeloid origin
NG2/Cspg4	Oligodendrocyte progenitors, Schwann cells, and perineurial cells
CD146	Immature endothelial cells, ganglion cells and activated T lymphocytes
Nestin	Progenitor cells
NeuroTrace 500/525	No reported expression
Astrocytes	
Marker	Non-astrocyte expression
GFAP	Progenitor cells, fibroblasts, enteric glia, satellite cells, Schwann cells, chondrocytes, myoepithelial cells, and lymphocytes
ALDH1L1	Neural stem cells
EAAT1/GLAST1	Microglia and oligodendrocytes
EAAT2/GLAST2	Microglia and oligodendrocytes
AQP4	Microglia
S100β	Oligodendrocytes, oligodendrocyte progenitor cells, and microglia
Aldolase C	Neurons
Microglia	
Marker	Non-microglial expression
CD11b	Monocytes, neutrophils, natural killer cells, granulocytes, macrophages, red blood cells, and memory B cells
Iba1	Macrophages, vascular smooth muscle cells, and neutrophils
CX3CR1	Monocytes, natural killer cells, circulating and skin resident dendritic cells, CD8+ T cells
CD45	All differentiated hematopoietic cells
Tmem119	Oligodendrocyte precursor cells
P2RY12	Granulocytes and Kupffer cells
FCRLS	Kupffer cells, Ileal macrophages, oligodendrocyte precursor cell, embryonic fibroblast
HexB	Hoffbauer cells and Kupffer cells

**Table 2:** Cre-LoxP mouse lines of the neurovascular unit

Cre mouse model	Marker	Known expression	Strengths	Limitations	References
Endothelial cells					
Tek-Cre/Tie2-Cre	Tie 2	EC, heart valves, and HC	Expression begins early and is consistent into adulthood	Expressed in HC	Payne et al. (2018)
Tg(Cdh5-cre/ERT2)^1Rha^	Cdh5	EC	Inducible and well characterized in retina and brain endothelium of post-natal mice	Reported variability in recombination	Kisanuki et al. (2001), Wang et al. (2010), Yanagida et al. (2017)
Tg(Cdh5-cre/ERT2)^#Ykub^	Cdh5	EC	Inducible and characterized in pre-natal mice	Reported variability in recombination	Kisanuki et al. (2001)
Tg(Cdh5-cre/ERT2)^CIVE23Mlia^	Cdh5	EC and HC	Inducible and characterized in pre-natal mice	Reported variability in recombination	Kisanuki et al. (2001)
Pericytes					
PDGFRβ-Cre	PDGFRβ	Pericytes, NPCs, glial cells, fibroblasts	Constitutive and inducible forms	Specificity	Cuttler et al. (2011), Foo et al. (2006)
PDGFRβ-P2A-CreER^T2^	PDGFRβ	Pericytes, NPCs, glial cells, fibroblasts	Inducible and validated in brain and retinal pericytes	Specificity	Cuervo et al. (2017)
Pericyte-CreER	PDGFRβ/NG2	Pericytes	Two promoter model of specificity	Challenging breeding schemes	Kisler et al. (2020), Nikolakopoulou et al. (2019)
Astrocytes					
hGFAP-CreERT2xAi14	GFAP	Astrocytes, Schwann cells, and sensory ganglia satellite cells	Constitutive expression in 50–89% of astrocytes	Specific strain and induction method can change specificity and expression levels	Park et al. (2018)
Aldh1L1-Cre/ERT2	Aldh1L1	Astrocytes and NSC	Inducible and no detectable expression in neurons	Early post-natal induction affects other cell types	Winchenbach et al. (2016)
Microglia					
CX3CR1-Cre	CX3CR1	Microglia, monocytes, DC’s, NK cells, T and B lymphocytes	Commonly available in constitutive and inducible mouse lines	Cannot distinguish microglia and macrophages and reported leakage into neurons	Goldmann et al. (2016), Jung et al. (2000), Wieghofer and Prinz (2016)
Tmem119-CreERT2	Tmem119	Microglia and oligodendrocyte precursors	Distinguishes microglia and macrophages	Not characterized in pathological conditions	Kaiser and Feng (2019)
P2RY12-CreER	P2RY12	Microglia, and in a small subset of CD206+Cx3cr1+heart, intestine, lung, and splenic cells	Distinguishes microglia and macrophages	Downregulation in inflammatory pathologies	McKinsey et al. (2020)
Hexb-CreERT2	HexB	Microglia	Distinguishes microglia and macrophages and enhanced in parenchymal microglia	No downregulation in studied pathologies	Masuda et al. (2020)

*EC* endothelial cells, *HC* hematopoietic cells, *NPC* neural progenitor cells, *DC* dendritic cells, *NK* natural killer cells
